# Identification of new regulatory genes through expression pattern analysis of a global RNA-seq dataset from a *Helicobacter pylori *co-culture system

**DOI:** 10.1038/s41598-020-68439-8

**Published:** 2020-07-13

**Authors:** Nuria Tubau-Juni, Josep Bassaganya-Riera, Andrew Leber, Victoria Zoccoli-Rodriguez, Barbara Kronsteiner, Monica Viladomiu, Vida Abedi, Casandra W. Philipson, Raquel Hontecillas

**Affiliations:** 1 NIMML Institute, Blacksburg, VA 24060 USA; 2 BioTherapeutics, Inc, Blacksburg, VA USA

**Keywords:** Immunology, RNA sequencing

## Abstract

*Helicobacter pylori* is a gram-negative bacterium that persistently colonizes the human stomach by inducing immunoregulatory responses. We have used a novel platform that integrates a bone marrow-derived macrophage and live *H. pylori* co-culture with global time-course transcriptomics analysis to identify new regulatory genes based on expression patterns resembling those of genes with known regulatory function. We have used filtering criteria based on cellular location and novelty parameters to select 5 top lead candidate targets. Of these, Plexin domain containing 2 (*Plxdc2*) was selected as the top lead immunoregulatory target. Loss of function studies with in vivo models of *H. pylori* infection as well as a chemically-induced model of colitis, confirmed its predicted regulatory function and significant impact on modulation of the host immune response. Our integrated bioinformatics analyses and experimental validation platform has enabled the discovery of new immunoregulatory genes. This pipeline can be used for the identification of genes with therapeutic applications for treating infectious, inflammatory, and autoimmune diseases.

## Introduction

Chronic bacterial infections are maintained by complex and dynamic host-bacterial interactions at the cellular and molecular level that dictate the outcome of the host immune response in favor of persistence. The phenotypic manifestation of these interactions results from the coordinated expression of sets of genes with overlapping functions that cooperate in modulating host responses. In addition to pathogen-associated molecular patterns (PAMPs) that signal infection and are associated with innate anti-bacterial and inflammatory responses, other lesser known bacterial components elicit compensatory immunoregulatory responses that when exploited by pathogens, can promote an accommodating tissue microenvironment that favors long-term bacterial colonization. For instance, *Mycobacterium tuberculosis* induces IL-10-driven regulatory responses that suppress effector mechanisms leading to chronicity of infection^1–4^. Therefore, the activation of host immunoregulatory mechanisms by certain bacterial species inhibit the effector immune response and prevent bacterial clearance.

*Helicobacter pylori* is highly specialized to colonize the human stomach niche^5,6^. The infection is chronic and affects more than 50% of the world’s population^7^. *H. pylori* infection is mostly asymptomatic; however, approximately 10% of carriers develop peptic ulcers^8,9^, and 1–3% gastric cancer^9^. Interestingly, *H. pylori* infection may be an important driver of systemic tolerance in asymptomatic individuals with an inverse correlation between the presence of this bacterium and the development of autoimmune diseases, asthma, esophageal adenocarcinoma and type 2 diabetes^10–14^. These conflicting implications in disease may stem from the relative dominance of effector versus regulatory components of the immune response following infection^15–18^. Altogether, long-term colonization suggests that the strong *H. pylori-*induced immunoregulatory responses can promote immune tolerance and shift inflammatory and effector responses. Therefore, *H. pylori* interaction with immune cells emerges as an ideal and unique system to explore the underlying molecular pathways that control both edges of the immune response and discover novel immunoregulatory mechanisms.

Macrophages have been described as key cellular contributors in *H. pylori-*induced regulatory mechanisms^19^. Particularly, *H. pylori* interacts with a subset of mononuclear phagocytes that promotes IL-10-driven regulatory responses facilitating immune tolerance and enabling optimal colonization of the gastric mucosa^20^. We demonstrated that macrophage peroxisome proliferator-activated receptor gamma (PPARγ), an anti-inflammatory transcription factor, was needed for the induction of the full spectrum of regulatory responses^20^. Additional macrophage-expressed genes (*Par1*, *HO-1*) play a similar role to PPARγ and contribute to keeping high levels of colonization while reducing pathology and disease^21,22^. Early after the initiation of the immune response, both macrophages and dendritic cells (DC) endure strong metabolic and transcriptional reprogramming to respond to infection. Cellular programs of gene expression are highly coordinated in time, with sets of genes with overlapping roles sharing similar expression pattern by being upregulated and downregulated simultaneously. The loss of a single hub gene at the interface of immunity and metabolism can affect the equilibrium of the whole system having a significant impact on the outcome of the response. Indeed, suppression or inactivation of just PPARγ, results in stronger inflammatory responses, while activation or enhanced expression leads to a more balanced response, maintained by activation of immunoregulatory pathways that control key metabolic events and limit the upregulation of inflammation-prone genes^20,23–25^.

In this study, we pursued the identification of genes with regulatory function using *H. pylori* for its known ability to induce regulatory responses. Our method involved the use of a high-resolution time-course transcriptomic analysis of co-cultures of wild-type (WT) or PPARγ-deficient bone marrow-derived macrophages (BMDM) with live *H. pylori* that provided detailed patterns of expression of genes with validated pro-inflammatory or regulatory roles. We used a bioinformatics analysis system to sort transcripts with kinetic behavior similar to known regulatory genes and used a filtering criterion that provided a short list of potential new immunoregulatory candidates. One of them, Plexin domain containing 2 (*Plxdc2*), was chosen and further confirmed to have an immunoregulatory role with the potential to be used as molecular target for the development of treatments for inflammatory and autoimmune diseases.

## Result

### Calibration of the BMDM-*H. pylori* co-culture in vitro system

**Figure 1:**
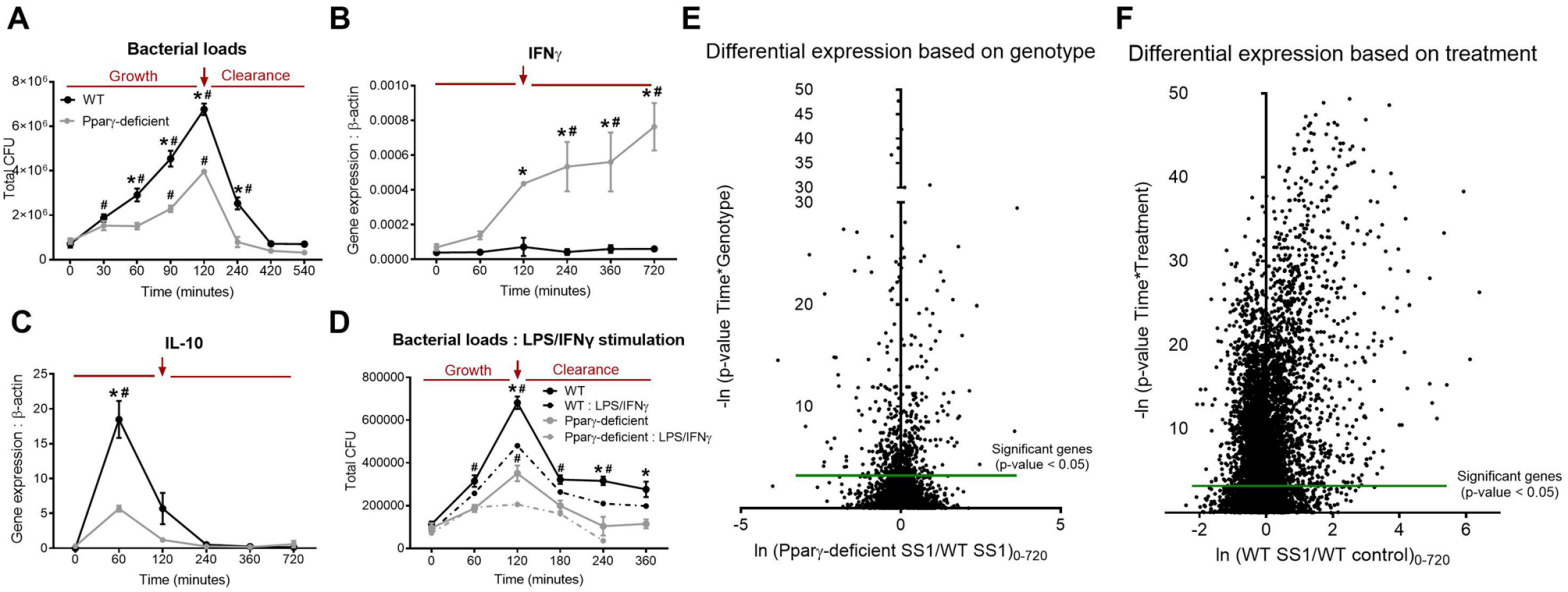
*Helicobacter pylori* co-culture alters macrophage transcriptomic profile, leading to the activation of early regulatory responses and increasing bacterial persistence in WT cells. WT and PPARγ-deficient BMDM were co-cultured ex vivo with *H. pylori* and cells were harvested at several time-points ranging from 0 to 720 min after gentamycin treatment. Bacterial burden (**A**) and gene expression, including IFNγ (**B**) and IL-10 (**C**) were assessed. To classically activate macrophages prior to *H. pylori* co-culture, cells were stimulated with LPS and IFNγ, then challenged with *H. pylori* and bacterial burden was measured (**D**). Differential gene expression based on genotype (**E**) and *H. pylori* infection (**F**) from a whole transcriptomic analysis performed on the harvested cells were also assessed. The red arrow highlights the 120 min timepoint that corresponds to the peak of bacterial growth. *P < 0.05 between genotypes, ^#^*P* < 0.05 within each genotype compared to time 0.

We sought to explore *H. pylori* interactions with BMDM employing a gentamycin protection co-culture system comparing cells obtained from WT and PPARγ-deficient (PPARγ fl/fl;LysCre+) mice. BMDM cultures were exposed to live *H. pylori* (strain SS1) for 15 min and then treated with gentamycin. Of note, *H. pylori* is internalized by macrophages and it can replicate in the intracellular compartment^26–29^. Therefore gentamycin was used to kill live extracellular bacteria after initial exposure and synchronize the cellular response. In addition, we used intracellular replication post-gentamycin treatment as a marker of the effect and status of the anti-bacterial response. In PPARγ-deficient mice, lack of this transcription factor results in defective expression of genes with regulatory function and overexpression of pro-inflammatory and anti-bacterial response genes^30–32^. Thus, the comparison between WT and PPARγ-deficient cells was used to assess altered expression of pro-inflammatory and regulatory programs. Cells were harvested at several time-points from 0 to 12 h post-gentamycin. We performed a preliminary analysis to calibrate timing of bacterial growth, peak and clearance along with the expression of pro-inflammatory and regulatory genes. Initial replication was first detected 30 min post-gentamycin treatment, peaked at 120 min in both genotypes and the bacteria were effectively cleared by 420 min (Fig. 1A). The kinetics were alike in both genotypes although bacterial counts in co-cultures of PPARγ-deficient macrophages were significantly reduced throughout the time course, starting at 60 min and up to 240 min post-challenge. This phenotype was compatible with an inflammatory shift due to the loss of PPARγ.

To overlay findings on bacterial replication with gene expression, we measured canonical pro-inflammatory and regulatory genes. IFNγ expression following *H. pylori* challenge was remarkably increased in PPARγ-deficient BMDM compared to WT (Fig. 1B). In contrast, WT macrophages upregulated the anti-inflammatory cytokine IL-10 at 60 min, that was significantly diminished in PPARγ-deficient BMDM (Fig. 1C). In addition, the IL-10 response overlapped with the phase of bacterial growth while IFNγ expression coincided in time with the phase of bacterial clearance. These data fit with a model of coordinated expression of an initial regulatory response during bacterial replication that is followed by a dominant inflammatory response required for bacterial clearance. To validate this assessment, WT and PPARγ-deficient macrophages were classically activated through LPS/IFNγ stimulation 24 h prior the infection. Generation of pro-inflammatory WT macrophages resulted in a suppression of *H. pylori* loads at the 120-min time point (Fig. 1D). Moreover, the bacterial burden peak was entirely abrogated in LPS/IFNγ-treated PPARγ-deficient cells. To elucidate the underlying regulatory and anti-bacterial mechanisms activated upon *H. pylori* co-culture, we performed a global transcriptomic analysis on the gentamycin protection assay time course (0, 60, 120, 240, 360 and 720 min). Initial RNAseq analysis demonstrated significant differences in the gene expression profile within both genotype (Fig. 1E) and treatment (Fig. 1F). Almost 50% of genes exhibited significant differential expression based on the treatment, with a general upregulation after *H. pylori* challenge. Thus, *H. pylori* influences the macrophage transcription profile, and these changes translate in modifications of macrophage function that favor the generation of a regulatory phenotype.

### Validation of experimental co-culture system with identification of differential expression patterns in characterized antimicrobial genes

**Figure 2:**
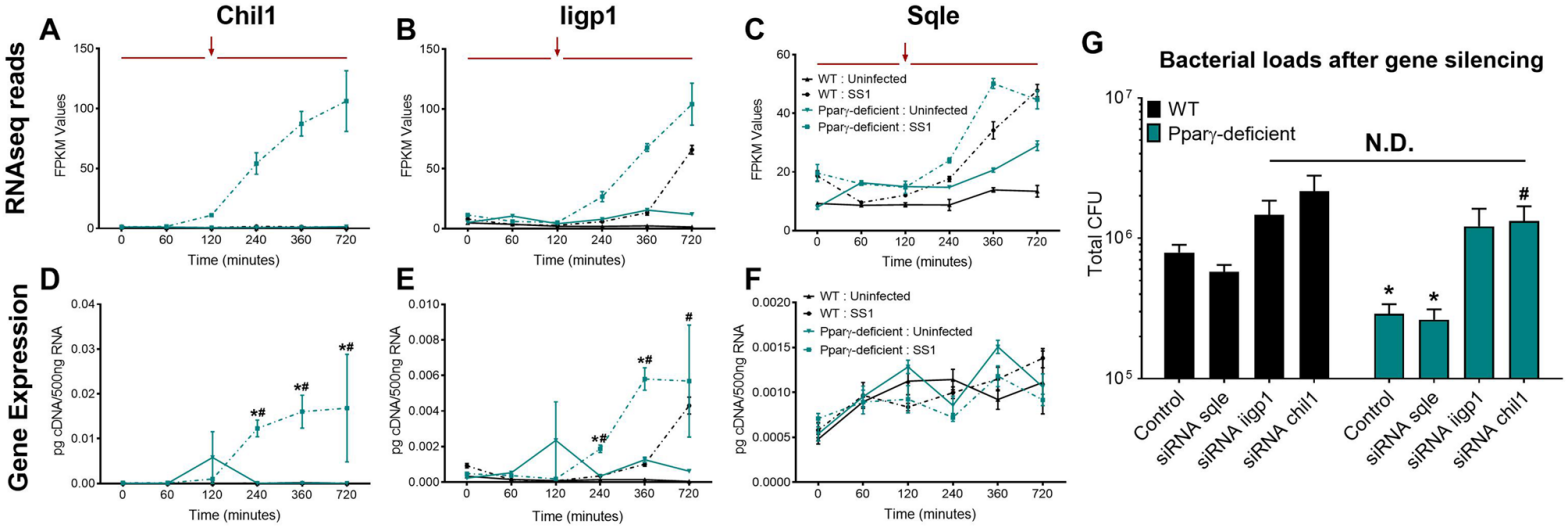
Initial analysis and validation of the whole transcriptomic analysis revealed two genes with differential expression pattern and well-defined anti-microbial functions. Plots represent RNAseq reads of *Chil1* (**A**), *Iigp1* (**B**), and *Sqle* (**C**) during the entire time-course comparing genotypes and treatments. *Chil1* (**D**), *Iigp1* (**E**), and *Sqle* (**F**) gene expression from the same co-culture was validated through qRT-PCR. Bacterial loads (**G**) were measured 120 min post-gentamycin treatment of *H. pylori* co-cultures in WT and PPARγ-deficient macrophages transfected with *Chil1*-targeted, *Iigp1*-targeted or *Sqle*-targeted siRNA or a scrambled sequence as a negative control. *P < 0.05 between genotypes, ^#^*P*-value < 0.05 within each genotype compared to time 0.

The expression of regulatory and pro-inflammatory genes is tightly regulated and coordinated over time. Indeed, as our co-culture system indicates, pro-inflammatory and regulatory genes frequently present opposite expression kinetics under the same conditions. We used the transcriptomic dataset to evaluate the expression of 9 established canonical pro-inflammatory genes (*Mcp1, Mcp5, IL6, IFN*γ*, IL12a, IL12b, Cxcl1, Cxcl10* and *Mip-1*α) to initially explore and identify distinctive patterns of expression associated with pro-inflammatory and antimicrobial functions. Expression of inflammation-related genes was delayed compared to regulatory genes, and induced around the peak of replication, at 120 min of exposure to live *H. pylori* (Supplementary Fig. S1). As expected, lack of PPARγ resulted in increased pro-inflammatory gene expression. We followed this initial evaluation by performing a 3-Way ANOVA for genotype, treatment, and time effects on the whole transcriptomic dataset. Eight genes, *Chil1*, *Etv5*, *Iigp1*, *Ptger4*, *Sqle*, *Osm*, *Rptoros* and *Hspa2,* were identified with *P* < 0.05. The interaction between these effects varied between genes but many were late-response genes that were significantly upregulated in PPARγ-deficient macrophages (Supplementary Fig. S2). This was consistent with the identified pro-inflammatory functions of many of these genes. We focused our validation analysis on *Chil1*, *Iigp1* and *Sqle* (Fig. 2A–C)*,* due to their expression kinetics that resemble the identified inflammatory gene patterns and their well-known role in host–pathogen responses. Validation by qRT-PCR confirmed the RNAseq expression pattern of *Chil1* and *Iigp1* (Fig. 2D,E). In contrast, the late upregulation of *Sqle* in the infected groups was undetectable (Fig. 2F). *Chil1* and *Iigp1* gene silencing (Supplementary Fig. S3) resulted in increased bacterial loads in both genotypes (Fig. 2G). As expected, there were no differences due to silencing of *Sqle*. Therefore, the initial analysis of this global transcriptomics dataset based on patterns of expression related to pro-inflammatory functions highlighted two genes, *Chil1* and *Iigp1*, with a relevant, previously established role in macrophage antimicrobial responses. This initial analysis validated the potential use of this system for the identification of function-linked transcripts based on the kinetic pattern of expression, and the use of a PPARγ-deficient BMDM to functionally subtract regulatory genes from the system.

### Pattern of expression-based analysis identified five candidates as novel genes with putative regulatory functions

To identify the initial set of reference expression patterns with regulatory functions, regulatory Nod-like receptors (NLRs) and PPAR canonical immune pathways, that encompass > 200 genes, were selected. NLRs are a subfamily of pattern recognition receptors, that similar to PPARs, regulate innate immune responses and metabolism in macrophages. Upon ligand binding, downstream activation of the NLRX1 pathway results in the initiation and regulation of potent inflammatory mechanisms^33,34^. These two canonical pathways in conjunction contain a mixture of genes with dominant pro-inflammatory or anti-inflammatory role, which facilitates the identification of regulatory patterns by comparison to transcripts of inflammatory genes.

**Figure 3:**
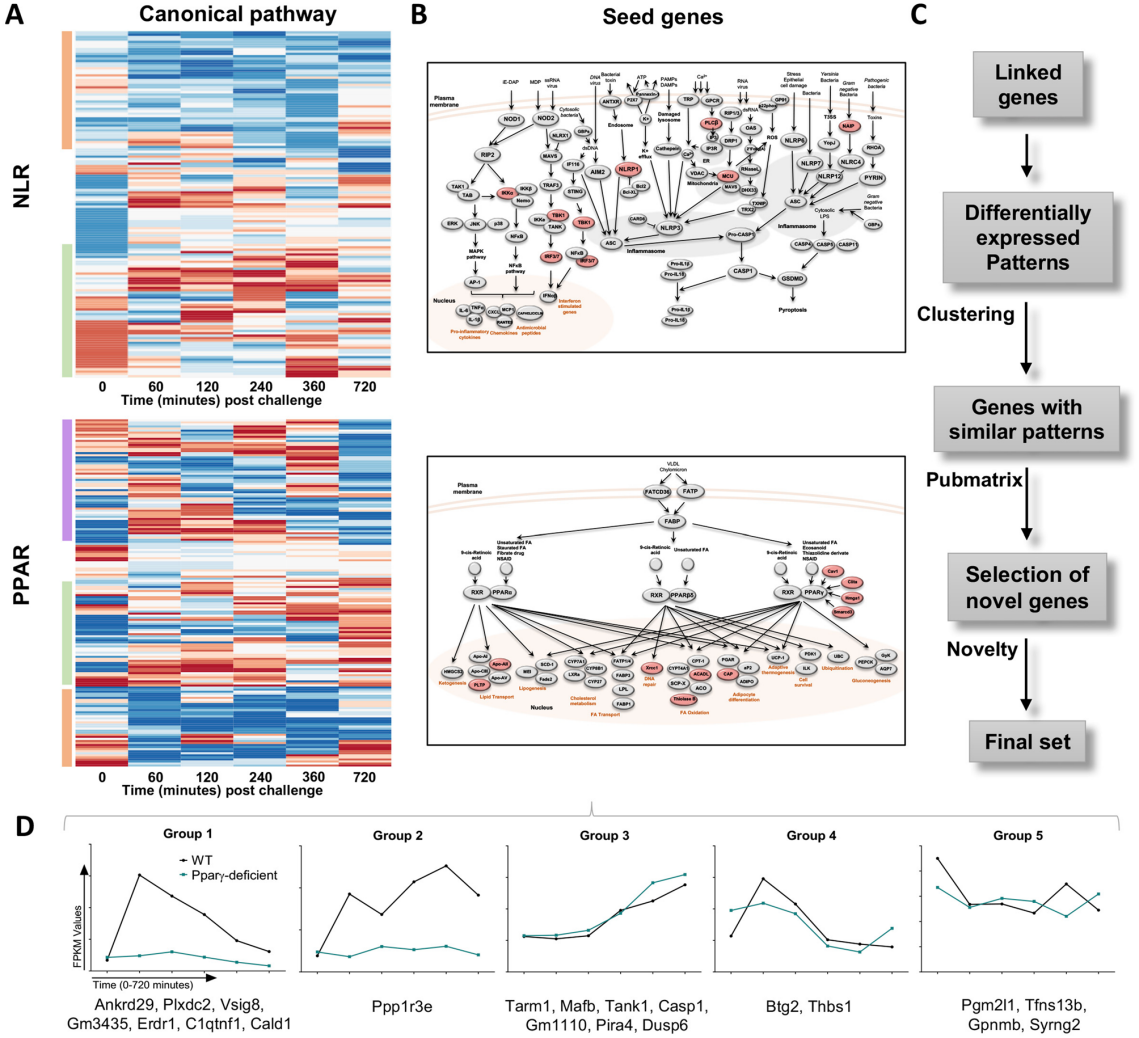
Bioinformatics pipeline utilized to analyze the RNAseq dataset and establish the differential expression patterns that lead to the identification of potential regulatory candidates. Heatmaps represent the genotype fold-change expression from the entire time-course (0–720 min post challenge) from each gene in the NLR and PPAR pathways (**A**). Blue represents inhibited expression in WT macrophages compared to the upregulation in PPARγ-deficient, while red indicates upregulation in WT compared to a suppressed expression in PPARγ-deficient macrophages. Heatmaps were generated with R software (version 3.2.3). NLR and PPAR pathways are represented in these diagrams (**B**). These images were generated with Microsoft PowerPoint (version 15.40). Top genes, based on the differential expression analysis represented in the heatmap, are highlighted in red and selected as seed genes. Schematic representation of the steps performed during the bioinformatics analysis, after the seed genes selection (**C**). The 21 genes included in the final set were classified in five groups based on their expression pattern (**D**).

Initially, we analyzed the WT gene expression fold change in comparison to PPARγ-deficient in both pathways across the entire time course and presented in the form of heat maps (Fig. 3A) where blue represents genes downregulated in WT compared to PPARγ-deficient and red represents upregulation of gene expression in WT related to PPARγ-deficient. Based on our assumption, inflammatory genes would fall within the WT downregulated (blue) cluster, while regulatory genes would be found within the WT upregulated (red) group due to the inverted patterns of regulatory and pro-inflammatory gene expression between genotypes. Indeed, the bioinformatics analysis revealed specific expression patterns in both signaling pathways that were clustered in groups. The NLR pathway includes two well-defined clusters based on genotype (Fig. 3A). The orange box, at the top, contains genes upregulated in PPARγ-deficient BMDM, and the green box, at the bottom, contains a second class of genes with greater expression in the WT group. Interestingly, the PPARγ-deficient upregulated genes had a delayed expression pattern, while WT upregulated genes presented an earlier peak. These two patterns were also represented in the PPAR pathway, depicted in orange and green boxes (Fig. 3A). The analysis of genes associated with PPAR revealed an additional third cluster, highlighted in purple, including a group of genes characterized by a random pattern among the entire time course. A plausible explanation is the existence of a strong PPARγ interaction with these genes. Therefore, the absence of the transcription factor in PPARγ-deficient BMDM could alter the expression of these genes, due to direct activation, inhibition or upregulation of compensatory mechanisms, that result in a fluctuating pattern.

Gene selection was narrowed to the green box cluster (i.e. genes upregulated in WT), to include transcripts with a positive response to *H. pylori* in WT BMDM prior to the peak of bacterial loads reported in this genotype at 120 min (Fig. 1A). The choice included 7 NLR and 10 PPAR pathway genes highlighted in red (Fig. 3B), which were defined as seed genes. Based on the expression patterns of the seed genes, we built an initial dataset that comprised these genes and a group described as pattern-linked genes obtained from the global transcriptome dataset. Linked genes are characterized by having similar kinetics to seed genes. A subset of differentially expressed patterns were selected in this large initial dataset, and through 2 cycles of clustering within the entire global transcriptional dataset, we obtained a specific group of genes containing potential candidates. To further narrow down our search, we utilized the Pubmatrix^35^ tool to select the most novel genes based on the following criteria: number of publications, cell location, and known function (Fig. 3C). For a final selection, the 21 genes included in the final set were divided in 5 groups based on their expression pattern (Supplementary Fig. S4). Groups 1 and 2 exhibited a clear distinctive pattern within genotype, whereas no genotype differences were observed in groups 3, 4 and 5 (Fig. 3D). Further, genes in groups 1 and 2 were upregulated post-*H. pylori* challenge in WT BMDM and suppressed in PPARγ-deficient, which we took as indication of potential regulatory function.

**Figure 4:**
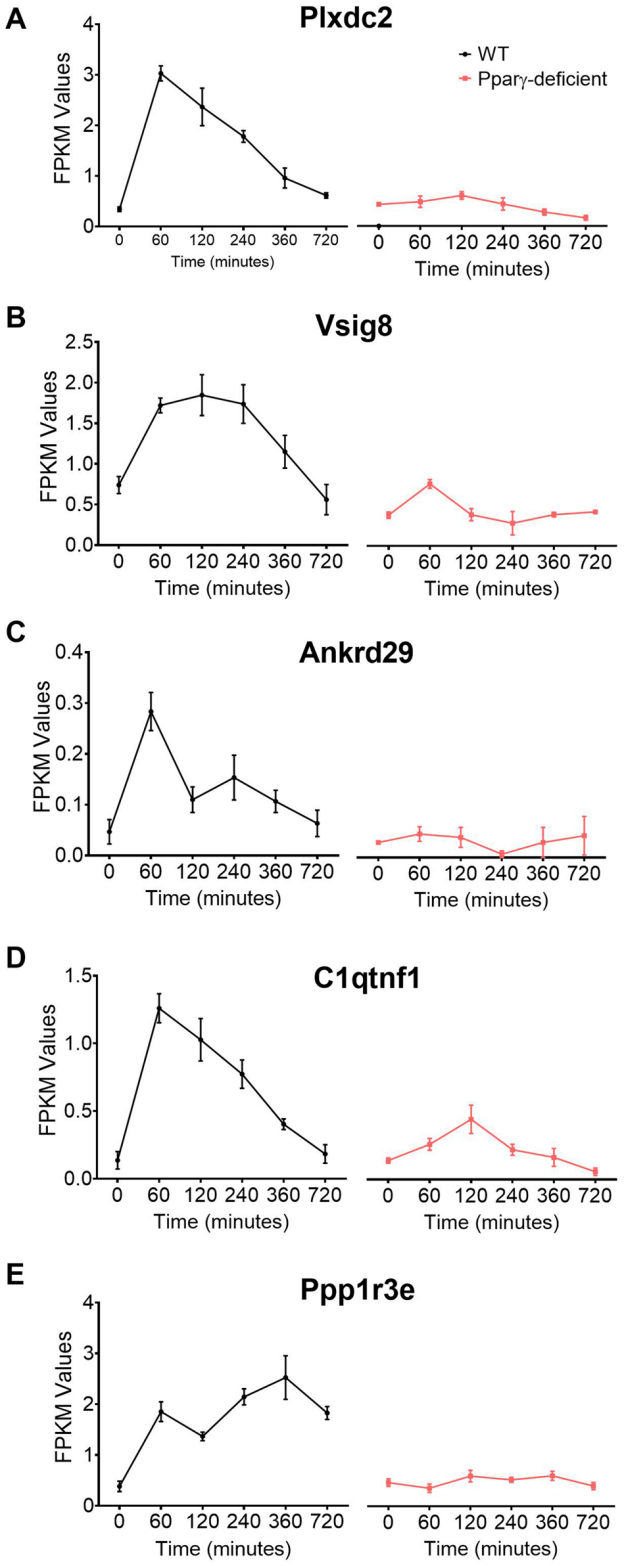
Expression kinetics of the five candidates selected from the bioinformatics analysis to undergo experimental validation. *Plxdc2* (**A**), *Vsig8* (**B**), *Ankrd29* (**C**), *C1qtnf1* (**D**), and *Ppp1r3e* (**E**), RNAseq reads in WT and PPARγ-deficient macrophages expressed as FPKM values.

Based on the pattern plus Pubmatrix analysis, five genes from groups 1 and 2 of the final dataset were identified as potential new regulatory leads for further validation. Plexin domain containing 2 (*Plxdc2*, Fig. 4A), V-set and immunoglobulin domain containing 8 (*Vsig8*, Fig. 4B), Ankyrin repeat domain 29 (*Ankrd29*, Fig. 4C) and C11 and tumor necrosis factor related protein 1 (*C1qtnf1*, Fig. 4D) share an early expression peak in WT macrophages, which was abrogated in PPARγ-deficient cells, and coincided with the bacterial burden spike in the gentamycin protection assay. The kinetics of Protein phosphatase 1 regulatory subunit 3E (*Ppp1r3e*, Fig. 4E) had a biphasic expression with an initial peak at 60 min, a grove coinciding with the maximum bacterial growth and a second peak at 360 min. In PPARγ-deficient BMDM, all 5 candidates were consistently downregulated and displayed as a flat line. Known properties of these five genes are described in Supplementary Table S1. Publications linked to each of the genes reveal a large diversity of established functions; however, association with the immune response was not reported for the majority of the genes. Thus, the limited number of publications together with the current established function of each gene support the novelty of their potential interaction with host immunoregulatory mechanisms.

### In vivo and in vitro *H. pylori-*induced upregulation of all five lead target candidates in WT mice is abrogated in macrophage-specific PPARγ null mice

**Figure 5:**
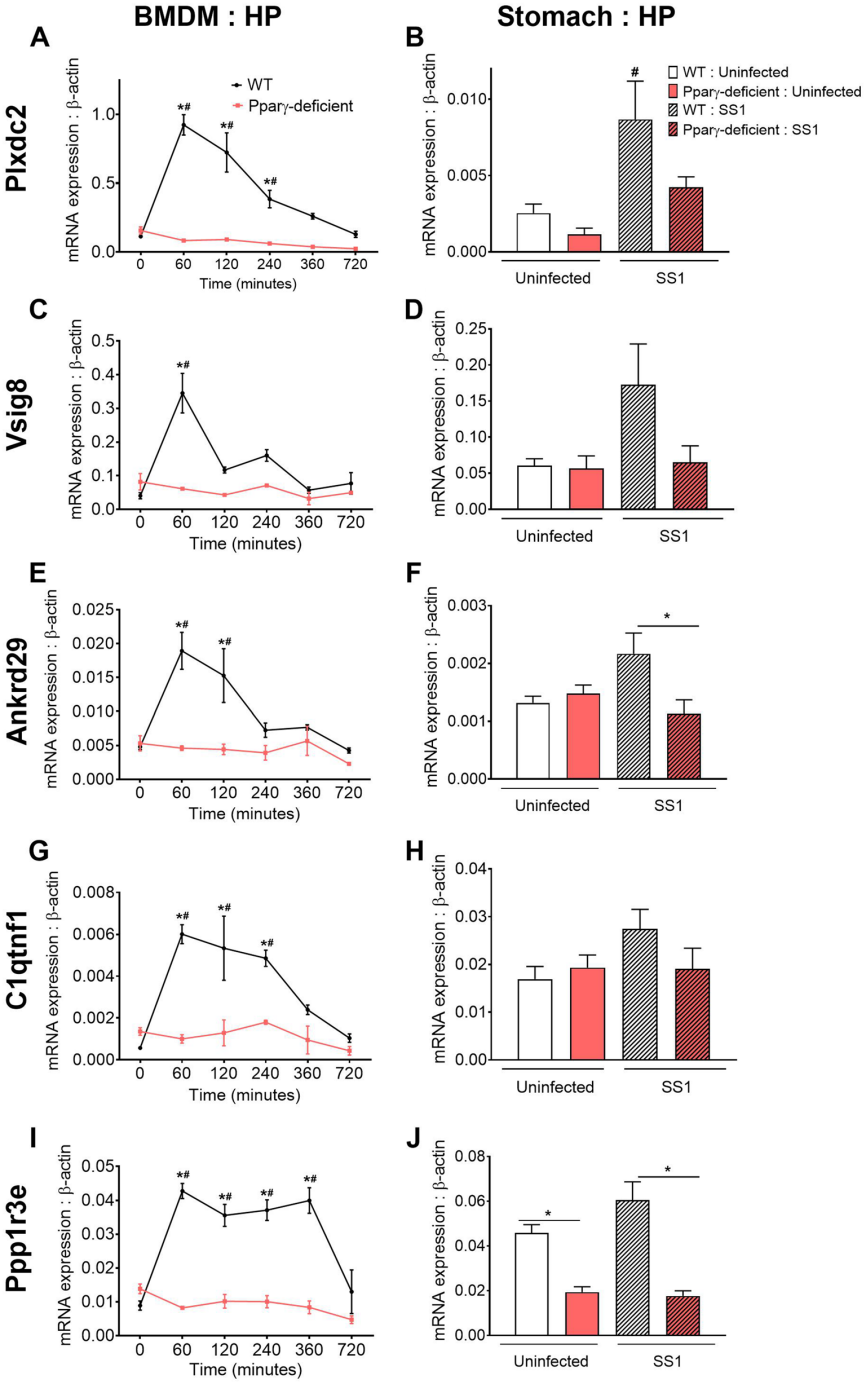
In vivo and in vitro validation of the five selected candidates under *Helicobacter pylori*-induced regulatory conditions. WT and PPARγ-deficient BMDM were co-cultured ex vivo with *H. pylori* and harvested at several time-points ranging from 0 to 720 min. *Plxdc2* (**A**), *Vsig8* (**C**), *Ankrd29* (**E**), *C1qtnf1* (**G**), and *Ppp1r3e* (**I**) gene expression was measured by qRT-PCR. WT and PPARγ-deficient mice were infected with *H. pylori* SS1 strain. Non-infected mice were used as control. *Plxdc2* (**B**), *Vsig8* (**D**), *Ankrd29* (**F**), *C1qtnf1* (**H**) and *Ppp1r3e* (**J**) gene expression was measured by qRT-PCR. *P < 0.05 between genotypes, ^#^*P*-value < 0.05 within each genotype compared to time 0.

To perform a validation of the five selected genes, we initially measured their expression by qRT-PCR on samples from each time point of the gentamycin protection assay used in the global transcriptomics analysis (Fig. 5A,C,E,G,I). All 5 genes presented a similar pattern to that observed in the RNAseq dataset, characterized by an early upregulation in WT BMDM that was suppressed in cells with a pro-inflammatory phenotype due to loss of PPARγ.

To explore the dynamics of the selected genes in vivo, WT and PPARγ-deficient mice were infected with *H. pylori.* Expression of the 5 candidate genes was measured in stomachs from infected and uninfected mice collected at day 28*.* In a previous study, we demonstrated that *H. pylori* infection in vivo induces strong regulatory mechanisms driven by IL-10-expressing myeloid cells starting at day 14 post infection and reaching maximum levels at day 28 post infection^20^. Consistent with the in vitro findings, *H. pylori* infection upregulated the expression of *Plxdc2* (Fig. 5B), *Vsig8* (Fig. 5D), *Ankrd29* (Fig. 5F), *C1qtnf1* (Fig. 5H) and *Ppp1r3e* (Fig. 5J) in WT gastric tissue. In contrast, minimal or no differences were reported upon *H. pylori* infection in macrophage-specific PPARγ-deficient mice. Thus, activation of regulatory responses after *H. pylori* challenge in WT mice correlates with an increased transcription of the five selected genes. However, PPARγ deficiency in myeloid cells abrogates this effect on the selected lead regulatory genes.

### Plxdc2 deficiency induces the generation of pro-inflammatory macrophages and reduces bacterial burden during *H. pylori* infection

**Figure 6:**
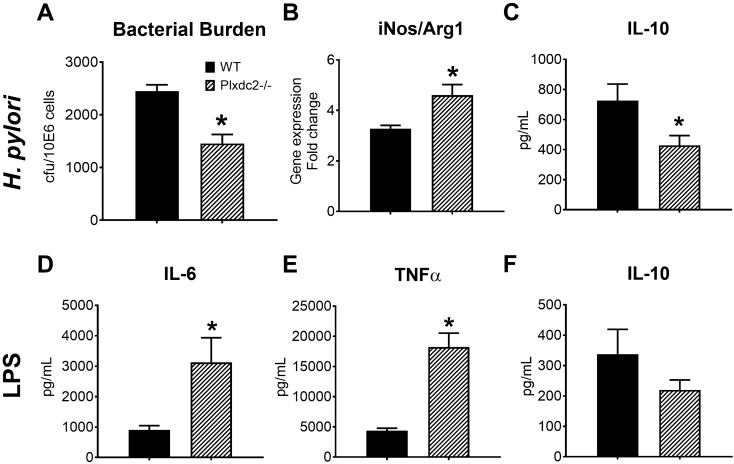
*Plxdc2* deficiency results into a macrophage pro-inflammatory switch in vitro*.* WT and *Plxdc2−/−* BMDM were co-cultured ex vivo with *H. pylori*. Bacterial burden (**A**) was measured 2 h post co-culture. iNOS/Arg1 gene expression ratio (**B**) was measured through qRT-PCR 6 h post co-culture. Accumulation of secreted IL-10 (**C**) in the culture supernatant was quantified through cytokine bead array 21 h post co-culture*.* WT and *Plxdc2−/−* BMDM were stimulated with 100 ng/mL of *E. coli* LPS and the secreted cytokine profile assessed by cytokine bead array 6 h post stimulation. Secreted IL-6 (**D**), *TNFα* (**E**) and IL-10 (**F**) were quantified. *P < 0.05.

Once initial screening and validation of the five lead candidates was completed, *Plxdc2* was chosen to further explore its regulatory activity. The selection was based on the reported expression kinetics, together with the fact that *Plxdc2* is a membrane receptor and a potentially druggable target. *Plxdc2*-deficient mice were utilized for these studies (Supplementary Fig. S5). BMDM obtained from WT and *Plxdc2−*/− mice were subjected to the gentamycin protection assay and harvested at 2, 6 or 21 h post co-culture. *Plxdc2* deficiency resulted in a twofold reduction of *H. pylori* burden compared to co-cultures of WT BMDM (Fig. 6A). To assess whether the reduced bacterial replication reported in *Plxdc2−/−* BMDM was due to modulation of macrophage function, we assessed the gene expression ratio between inducible nitric oxide synthase (*iNOS*) and Arginase 1 (*Arg1*). *Plxdc2*-deficient cells had increased iNOS/Arg1 ratio (Fig. 6B), a marker of pro-inflammatory phenotype. Additionally, IL-10 secretion (Fig. 6C) was significantly decreased in *Plxdc2*−/− cells. Similar results were obtained through *Plxdc2* gene silencing via siRNA studies with WT and PPARγ-deficient BMDM (Supplementary Fig. S6). *Plxdc2* silencing (70% of efficiency in WT BMDM, Supplementary Fig. S6A) prevented the early *Arg1* upregulation detected in the WT control group after *H. pylori* challenge (Supplementary Fig. S6B). In correlation with the gene expression results at 2 h post co-culture, *Plxdc2* silencing resulted in a threefold reduction of *H. pylori* burden in WT BMDM (Supplementary Fig. S6C). No differences were observed within the PPARγ-deficient groups. To further validate the regulatory function of *Plxdc2* in a different in vitro system, WT and *Plxdc2−/−* BMDM were stimulated with *E. coli* LPS (100 ng/mL), to assess cytokine profiles. Loss of *Plxdc2* resulted in a > threefold increase of IL-6 (Fig. 6D) and TNFα (Fig. 6E), and reduced IL-10 secretion (Fig. 6F). Therefore, *Plxdc2* deficiency leads to a pro-inflammatory shift in macrophage phenotype that abrogated *H. pylori*-induced regulatory responses, limiting bacterial persistence and replication.

### Plxdc2 deficiency is associated with increased inflammation and lower bacterial colonization in an in vivo model of *H. pylori* infection

**Figure 7:**
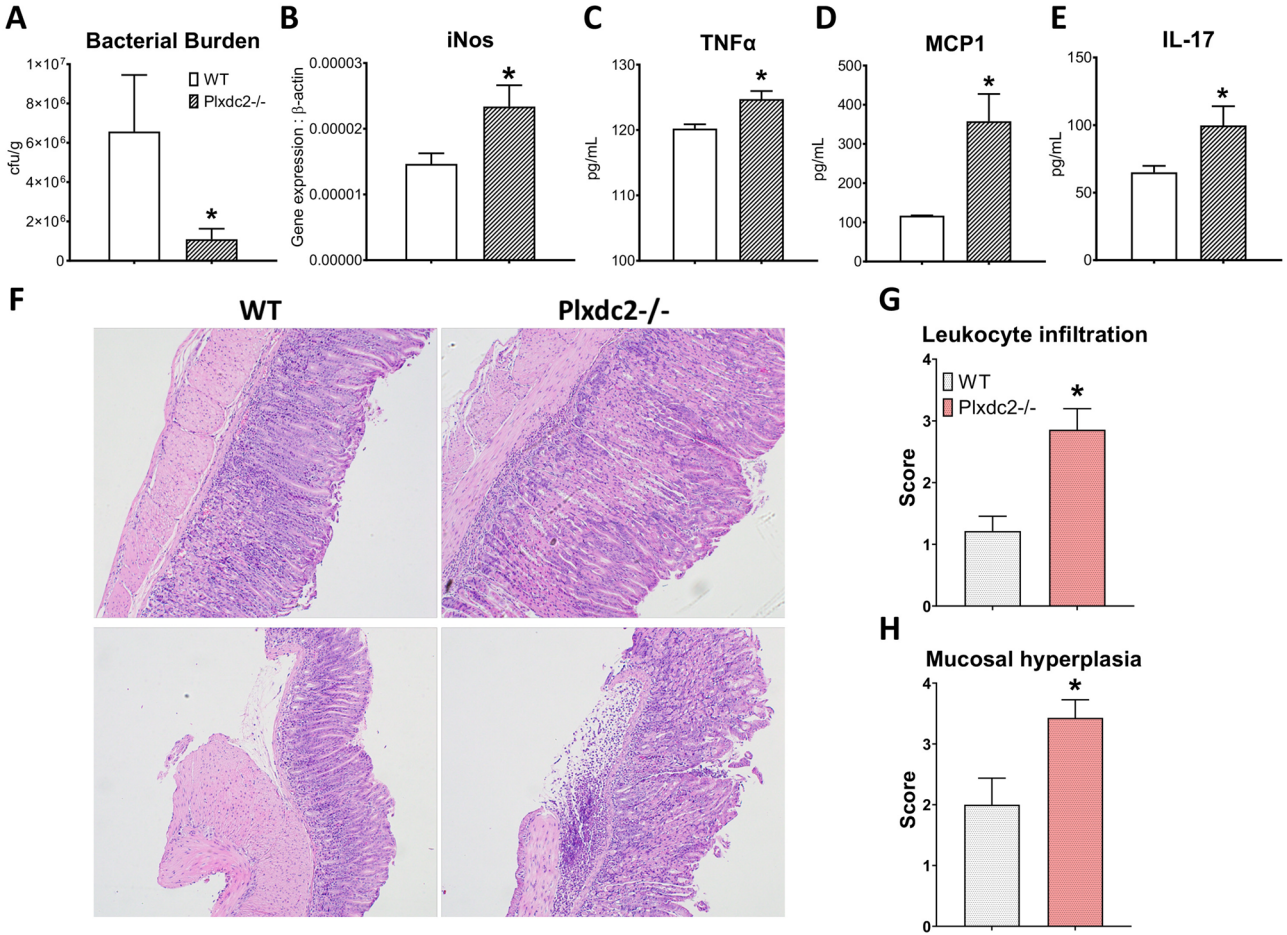
In vivo validation of *Plxdc2* regulatory role in a murine model of *H. pylori* infection. WT and *Plxdc2−/−* mice were infected with *H. pylori* SS1. Stomach and GLN were collected at day 28 post infection. Bacterial burden (**A**) and iNOS (**B**) expression were measured in gastric tissue. GLN-isolated cells were stimulated ex vivo with inactivated *H. pylori* SS1 for 72 h. Secreted TNFα (**C**), MCP1 (**D**) and IL-17 (**E**) were quantified 72 h post culture through cytokine bead array. WT and *Plxdc2−/−* mice were infected with *H. pylori* PMSS1. Representative images of hematoxylin and eosin-stained gastric sections WT and *Plxdc2−/−* mice infected with PMSS1 at day post infection 28 (**F**). These images were obtained using the Olympus CellSens Entry imaging software. Averaged histopathological scores of leukocyte infiltration (**G**) and mucosal hyperplasia (**H**) *P < 0.05.

To assess the role of *Plxdc2* in *H. pylori*-induced regulatory responses in vivo*,* WT and *Plxdc2−/−* mice were infected with 5 × 10^7^ cfu of *H. pylori* SS1. Similar to the in vitro results, *Plxdc2* deficiency significantly reduced bacterial colonization at day 28 post infection (Fig. 7A). Additionally, the decrease in bacterial loads correlated with increased gastric expression of iNOS (Fig. 7B). To assess whether *Plxdc2* deficiency shaped downstream antigen-specific responses, single-cell suspensions of gastric lymph nodes were co-cultured ex vivo with inactivated *H. pylori* SS1. Secreted TNFα (Fig. 7C), MCP1 (Fig. 7D) and IL-17 (Fig. 7E) were measured 72 h post stimulation and observed to be increased in the *Plxdc2*-deficient group. Therefore, our data suggests that *Plxdc2* expression contributes to the generation of *H. pylori*-driven regulatory responses during bacterial colonization. To assess if the loss of PLXDC2 plays a role in the induction of gastritis, WT and *Plxdc2−/−* mice were infected with 5 × 10^7^ cfu of *H. pylori* PMSS1. We chose PMSS1 because this strain colonizes the stomach more efficiently than SS1 and induces stronger immune responses. At day 28 post-infection *Plxdc2−/−* mice had a significant increase in inflammatory cells infiltrating the gastric mucosa and submucosa (Fig. 7F,G), which was accompanied by more severe mucosal hyperplasia (Fig. 7H) in comparison to the WT group. Of note, many uninfected *Plxdc2*-deficient mice displayed mild spontaneous gastritis (data not shown), which points towards the role of *Plxdc2* in maintaining tissue homeostasis.

### Loss of *Plxdc2* exacerbates disease severity and colonic inflammation in a DSS-induced model of colitis

**Figure 8:**
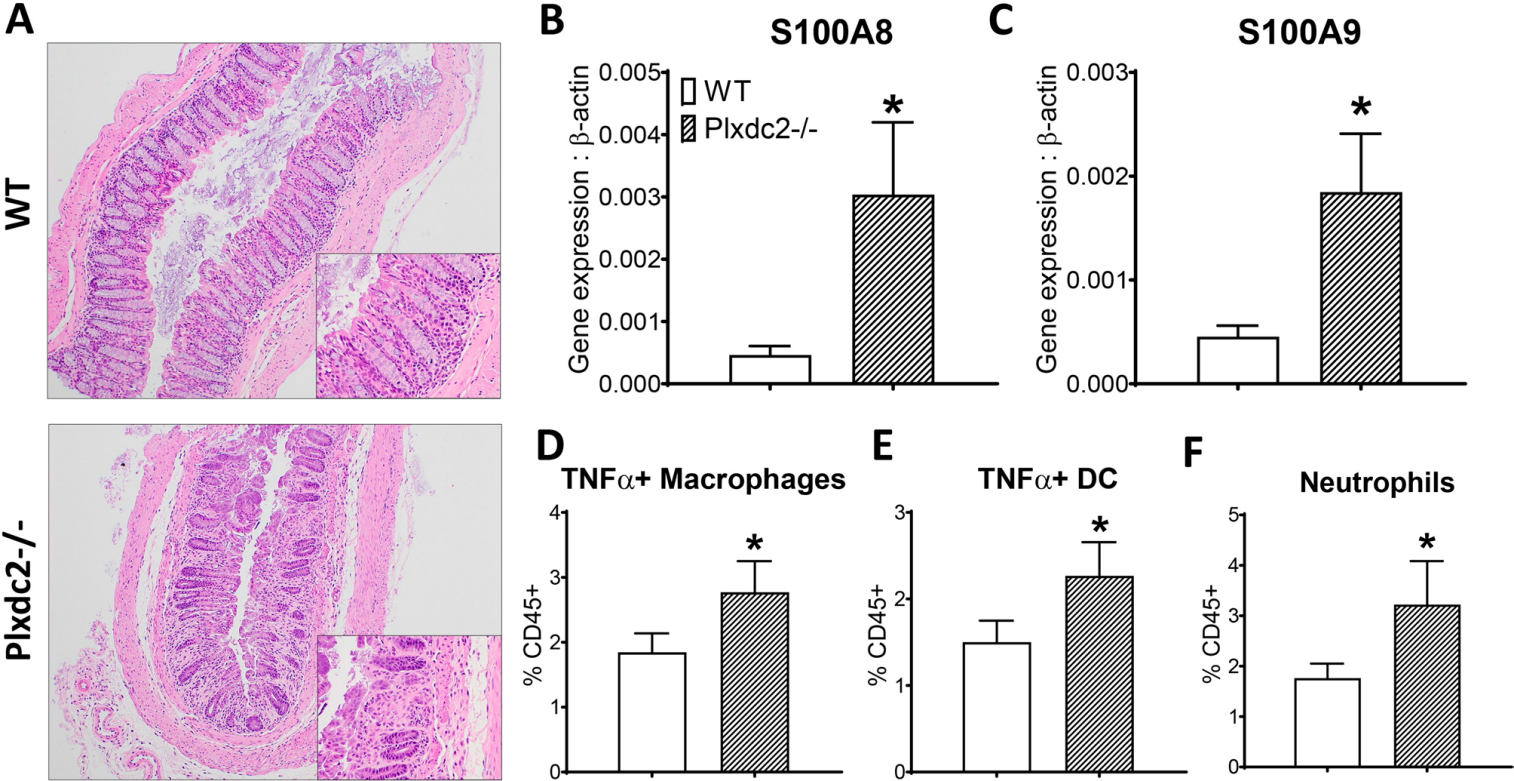
In vivo validation of *Plxdc2* as a novel regulatory candidate in a DSS model of colitis. WT and *Plxdc2−/−* mice were administered DSS in drinking water for a 7-d period. Representative images of H&E-stained sections of colonic tissue harvested on day 7 post-DSS challenge (**A**). These images were obtained using the Olympus CellSens Entry imaging software. Colon S100A8 (**B**) and S100A9 (**C**) expression was quantified through qRT-PCR. Percentages of colonic lamina propria infiltrating TNFα + Macrophages (**D**), TNFα + dendritic cells (**E**) and neutrophils (**F**) were assessed through flow cytometry analysis. *P < 0.05.

To further validate the regulatory role of *Plxdc2 *in vivo, WT and *Plxdc2−/−* mice were subjected to the DSS chemically-induced model of colitis. Histopathological analysis of colon tissue was conducted 7 days post-DSS challenge, at the peak of inflammation. *Plxdc2* deficiency resulted in increased leukocyte infiltration, mucosal thickening and epithelial erosion in comparison to the WT group (Fig. 8A). Expression of S100A8 (Fig. 8B) and S100A9 (Fig. 8C) genes, that encode for the neutrophil protein complex calprotectin, a well-established marker of inflammation and response to treatment in IBD, were also upregulated in *Plxdc2−/−* colons. Additionally, colonic lamina propria infiltration of pro-inflammatory macrophages (Fig. 8D), dendritic cells (Fig. 8E) and neutrophils (Fig. 8F) was also augmented in *Plxdc2−/−* mice. Therefore, this data indicates an elevated susceptibility to DSS challenge in *Plxdc2-*deficient mice, detected by an increase of colonic inflammation and overall disease severity associated with the loss of *Plxdc2.*

## Discussion

We present a novel system that leverages the regulatory responses induced by *H. pylori* in an ex vivo co-culture in combination with bioinformatics analyses to discover genes with potential immunomodulatory properties based on expression pattern kinetics in WT and PPARγ-deficient BMDM. Using this new platform, we have identified 5 candidate immunoregulatory genes: *Plxdc2*, *Ppp3e1r*, *Vsig8*, *Ankrd29* and *C1qtnf1*. The predicted regulatory role of the selected genes was further validated during an *H. pylori* challenge in vivo as well as through in vitro assays. Functionally, the correlation between the expression of the 5 candidates and the induction of regulatory mechanisms during *H. pylori* infection, support their potential immunomodulatory role. Moreover, the validation of the co-culture system provides new insights into the application of bioinformatics screening methods for the discovery of novel molecular targets for treating inflammatory and autoimmune diseases.

*Helicobacter pylori* plays an important role in the etiology of gastric pathologies, namely peptic ulcer and gastric cancer^36,37^. This gram-negative bacterium has long been known for its ability to induce mixed proinflammatory and immunoregulatory responses. Research on the latter has revealed several mechanisms that have been traced to specific bacterial components, such as low ability of LPS to activate TLR4 or VacA’s effect on inducing tolerogenic responses upon contact with dendritic cells, among others^38–40^. The simultaneous activation of these mechanisms is believed to contribute to a state of immune evasion or deviation that allows for chronic colonization of the human gastric mucosa. Here, we have leveraged *H. pylori’s* ability to induce regulatory responses to identify novel genes that participate in this response. We have used a simplified in vitro system with BMDM to avoid the complexity of the response in vivo where several cell types are involved. In the tightly controlled environment of the ex vivo co-culture with BMDM, *H. pylori* significantly influenced the gene expression profile. Because the method we describe depends on the analysis of kinetic patterns of gene expression, the use of a BMDM culture provided the additional advantage of response synchronization. We demonstrated that this synchronized ex vivo system together with the utilization of next generation sequencing (NGS) strategies and bioinformatics analysis, is a suitable approach to capture the full-spectrum of regulatory responses induced by *H. pylori* and to discover novel regulatory mechanisms with meaningful impact on the modulation of the immune response in vivo.

In addition to the combined NGS and bioinformatics analyses methods, in the current study, gene selection is based on the similarities between expression kinetics of candidate genes and known host genes with validated regulatory functions, to identify novel genes with similar characteristics. In our system, the comparison within macrophages with distinctive immunological steady states (WT versus PPARγ-deficient) was utilized to confirm modulation of sets of established genes and to validate the functional importance of new regulatory gene candidates.

To identify the predicted regulatory patterns, we utilized the canonical immune pathways of NLRs and PPARs. PPARs are nuclear receptors that regulate glucose and lipid metabolism and exert potent immunomodulatory functions. Indeed, activation of members in the PPAR pathway result in increased beta-oxidation of lipid metabolism and suppression of the inflammatory response^41^. The NLR family of 23 pattern recognition receptors is implicated in the initiation of innate immune responses in macrophages. As expected, NLR pathways include an important number of inflammation-driven genes^34,42,43^, but also genes with immunoregulatory functions, including NLRP10 and NLRX1^44–47^. Other pathways essential for the activation and maintenance of the immune response in macrophages were also considered, including the Toll-like receptors (TLR), the nuclear factor kB (NF-kB) and pathways associated with the production of reactive oxygen species (ROS). However, the strong association of such pathways with the initiation and amplification of inflammatory mechanisms with limited pro-regulatory functions in macrophages^48,49^, limited their potential value in our regulatory gene discovery pipeline.

Molecular evidence in vivo and in vitro suggests that *Plxdc2* and *Ppp1r3e* are the two genes with greatest immunomodulatory potential. *Plxdc2* encodes a 350-amino acid plasma membrane protein known to participate in cell proliferation and differentiation control during the development of the nervous system^50,51^. Our results suggest that the loss of *Plxdc2* alters macrophage phenotype in vitro causing a shift towards a more pro-inflammatory state, leading to a significant decrease of bacterial burden and downregulated expression of tissue healing and anti-inflammatory associated genes. *Plxdc2*, together with its homologue gene *Plxdc1*, has been identified as one of the membrane receptors of pigment epithelial-derived factor (PEDF)^52^, an endogenous anti-angiogenesis factor^53^. Interestingly, PEDF treatment modulates macrophage activation in Raw 264.6 cells through the increase of IL-10 production with potential association with PPARγ activation^54,55^. Cheng et al., reported that PEDF-induced IL-10 production in Raw 264.7 macrophages is dependent on both Plxdc1 and Plxdc2^52^. Our data not only suggests that *Plxdc2* can induce immunological changes independently of the external stimulation of PEDF, but it also indicates that *Plxdc2* expression is required for the efficient generation of *H. pylori-*driven regulatory responses in vivo*.* Indeed, loss of *Plxdc2* is associated with a decrease in bacterial colonization, together with an overall upregulation of pro-inflammatory markers and more severe gastric inflammatory pathology during *H. pylori* infection*.* The changes observed in the dynamics of *Plxdc2* over the time course and the gastric microenvironment switch during the in vivo infection, suggest additional signaling mechanisms with impact on anti-bacterial and overall immune responses. Moreover, the loss of *Plxdc2* significantly worsens experimental inflammatory bowel disease (IBD) caused by the DSS model of colitis, based on an exacerbated disease severity, inflammatory cytokine expression, and more severe colonic lesions in *Plxdc2−/− *mice.

The established regulatory effect of PEDF in macrophages, together with the cellular location of PLXDC2 and the potential for target druggability, provided a rationale for the selection of *Plxdc2* for further validation using knockout mice. However, the observed kinetics of *Ppp1r3e* also support the regulatory role of this gene and encourage its further investigation. *Ppp1r3e* gene encodes an insulin-regulated glycogen-targeting subunit for the protein serine/threonine phosphatase 1 (PP1), a protein involved in the regulation of several important cell functions, including gene expression, metabolism and cell death^56,57^. Glycogen-driving subunits increase glycogen production promoting glycogen synthase activity^56^. Glycogen plays a role in the metabolic interplay of immune cells^58^. Dendritic cells use glycogen as a fuel during the initiation of the immune response^58^. Deficiencies in glycogen metabolism alter the proper activation of dendritic cells^58^. Activated macrophages, with overexpression of the glucose transporter GLUT1 and increased glucose consumption, upregulate glycogen synthesis^59^. Therefore, through the modulation of glycogen metabolism, *Ppp1r3e* might modulate the activation of immune cells.

In conclusion, this study describes a new method of identifying candidate therapeutic targets with promising regulatory and immunometabolic roles. The main criteria used for candidate selection were (1) early upregulation coinciding with the phase of *H. pylori* growth, (2) blunted expression in PPARγ-deficient BMDM. Our pipeline was designed to sort genes with these properties although we cannot rule out the existence of regulatory genes with different kinetics of expression in our time course dataset. While 5 new targets were identified, initial validation studies supported *Plxdc2* and *Ppp1r3e* as two genes with great potential to modulate immune and host responses. Additional in vivo loss of function studies confirmed *Plxdc2* regulatory function and its ability to shape the host immune response during infection with *H. pylori* and in a mouse model of IBD. Thus, our screening platform can provide new insights in the identification of novel therapeutic targets for the modulation of the immune response. As a part of new drug development programs, these novel targets can drive the next wave of first-in-class therapeutics for autoimmune diseases.

## Materials and methods

### Animal housing and ethic statement

6 to 10 week old C57Bl/6J wild-type (WT), PPARγ fl/fl;LysCre+ (PPARγ-deficient) and *Plxdc2−/−* mice were used for these studies. PPARγ-deficient mice were generated by breeding PPARγ fl/fl into Lys-Cre mice, to produce mice lacking PPARγ in myeloid cells. *Plxdc2−/−* are whole body knock outs. All mice were bred and housed in the same colony in ventilated racks and under a 12-h light cycle. Mice were euthanized with CO_2_ narcosis followed by cervical dislocation as a secondary method. All experimental procedures performed were approved by the Institutional Animal Care and Use Committee (IACUC), met or exceeded requirements of the Public Health Service/National Institutes of Health and Animal Welfare Act and were conducted according to the approved guidelines and regulations.

### Bone marrow-derived macrophage (BMDM) isolation and culture

Bone marrow-derived macrophages (BMDM) were isolated as previously described^60^. Briefly, bone marrow (BM) was flushed out from sterilized femur and tibia of euthanized mice. Osmotic lysis was used to remove red blood cells. Samples were differentiated in cRPMI (RPMI 1640, 10% Fetal bovine serum, 2.5% Hepes, 1% Sodium pyruvate, 1% l-glutamine, 1% Penicillin/Streptomycin and 50 uM β-mercaptoethanol) supplemented with 25 ng/mL of recombinant mouse macrophage colony-stimulating factor (M-CSF) at 37 °C, 5% CO_2_ and 95% humidity. At day 3 fresh M-CSF-supplemented media was added. On day 6, BMDM were harvested. Cells were re-suspended in cRPMI and left to adhere overnight at 37 °C, 5% CO_2_ and 95% humidity.

### *H. pylori* culture and preparation of the inoculum

This study was performed using *H. pylori* SS1 and PMSS1 strains. *H. pylori* was cultured at 37 °C under microaerophilic conditions in Difco Columbia blood agar plates supplemented with 7% of Horse laked blood and *H. pylori* selective supplement (5 mg of Vancomycin, 2.5 mg of Trimethoprim, 2.5 mg of Cefsulodin and 2.5 mg of Amphotericin B). For in vivo inoculum preparation, *H. pylori* was harvested in 1 × PBS and adjusted to 2.5 × 10^8^ colony forming units (cfu) per mL, that corresponds to an optimal density (OD) of 1.2 at a 600-nm wavelength. For in vitro inoculum preparation, *H. pylori* was harvested in antibiotic-free cRPMI and adjusted to 1 × 10^8^ cfu/mL as described above.

### Gentamycin protection assay

BMDM cells were washed and fresh antibiotic-free cRPMI was added to the plates. Cells were infected with *H. pylori* SS1 at MOI 10 and synchronized by quick spin to ensure immediate contact. After a 15-min incubation, non-internalized bacteria were killed by thoroughly washing the cells with PBS/5% FBS containing 100 ng/mL Gentamycin. Time 0 cells were washed and immediately collected for downstream assays. The cells allocated for the remaining time points (30 min to 12 h) were covered with culture media until collection for downstream analysis.

### Bacterial re-isolation from BMDM and stomachs

BMDM were washed 3 times with sterile 1 × PBS. Cells were detached using a cell scraper in 200 μL of Brucella broth. Cell suspensions were sonicated 5 s to release intracellular bacteria. Stomachs were collected in 200 μL of Brucella Broth and homogenized using a grinder.

Serial tenfold dilutions of the original homogenates (from stomachs or BMDM) were plated onto the *H. pylori* plates described above. Colonies were counted 4 days after culture.

### Gene expression

BMDM cells were collected in ice-cold RLT (supplemented with β-mercaptoethanol). Excised stomachs were longitudinally opened, rinsed twice in 1 × PBS and stored in RNAlater. Total RNA was extracted from BMDM and stomach using the RNeasy mini kit (Qiagen) following the manufacturer’s instructions. RNA concentrations were quantified on a Nanodrop. iScript cDNA synthesis kit (Bio-Rad) was utilized to generate cDNA from RNA samples. Standard curves were generated by a series of dilutions from known concentration of purified primer-specific amplicon generated by PCR using Taq Polymerase. Total gene expression levels were assessed through a quantitative real-time PCR (qRT-PCR) using a CFX96 Thermal Cycler and SYBR Green Supermix (Bio-Rad). Beta-actin expression was utilized to normalize the expression levels of target genes. Primer sequences are included in the supplementary information.

### Global transcriptome sequencing

RNA isolated from WT and PPARγ-deficient BMDM collected at time-points 0, 30, 60, 120, 240, 360 and 720 min post-infection was submitted for whole transcriptome gene expression analysis using Illumina Hiseq. Once fastq files containing 100 bp-long pair-end reads were received, poor quality reads (> 40% of bases with PHRED score < 10; percentage of N greater than 5%; and polyA reads) were excluded. Through the utilization of Bowtie^61^ (version: 1.0.0) with parameters set to ‘-l 25 -I 1 -X 1,000 -a -m 200’, the remaining reads were mapped to RefSeq (mm10 from https://genome.ucsc.edu/). To calculate gene expression levels we used RSEM^62^, a program based on expectation–maximization algorithm. FPKM^63^ (fragments per kilobase per million sequenced reads) was used as the measurement of expression level. Data was submitted to NCBI's GEO database (Accession Number GSE67270).

### Bioinformatics analysis

As described in Fig. 3C, an initial dataset of genes linked to the selected NLR and PPAR candidates was generated. The Genome-scale Integrated Analysis of gene Networks in Tissues (GIANT) and Gene Expression Omnibus (GEO) databases were used and integrated in the Ingenuity Pathway Analysis (IPA) software to build the initial group of genes. Hierarchical based clustering was employed to obtain differentially expressed patterns within the combined initial NLR and PPAR genes and the linked dataset generated. The *hclust* method with Ward’s minimum variance method and Manhattan distance metric in R were used to cluster the data. Another clustering cycle was performed in order to obtain a larger set of genes with similar patterns of interest. The generated dataset was enhanced for novelty using the Pubmatrix tool, and a final dataset of genes was obtained.

### Gene silencing and BMDM stimulation

WT and PPARγ-deficient BMDM were transfected with 20 nM siRNA (27mer Dicer-substrate siRNA, DsiRNA), for target gene or scrambled sequence as a negative control (Integrated Device Technology) using Lipofectamine RNAiMax Transfection Reagent (Thermo Fisher Scientific) 48 h before infection. Media was replaced 6 h post-transfection. Gene knock-down was validated by qRT-PCR.

For the induction of a pro-inflammatory environment, BMDM were pre-treated with *E. coli* LPS (100 ng/mL) and rIFNγ (100 ng/mL) overnight. For LPS stimulation, BMDM were treated with *E. coli* LPS (100 ng/mL) for 6 h.

### In vivo *H. pylori* infection

8 to 10-week-old WT, PPARγ-deficient, and *Plxdc2−/−* mice were administered 200 μL of freshly prepared 5 × 10^7^ cfu of *H. pylori* SS1 or PMSS1 in 1 × PBS through orogastric gavage on days 0 and 2. A non-infected group administered with 200 μL of 1 × PBS without bacteria was also included. Mice were monitored for signs of disease weekly and stomach samples were collected at day 28 post infection.

### Gastric lymph node (GLN) isolation and ex vivo culture

GLN were excised and subjected to enzymatic digestion for 1 h at 37 °C in RPMI media supplemented with Collagenase and DNAse. 2 × 10^5^ cells were plated in 96-well plates and stimulated with 5 ng/mL of inactivated SS1 antigen. Samples were incubated for 72 h at 37 °C 5% CO_2_ 95% humidity. Supernatants were collected and the secreted cytokine profile analyzed using a Cytokine Bead array (BD Biosciences) following the manufacturer’s instructions.

### Dextran-sodium sulfate (DSS) colitis-induced model

8 to 10-week-old WT and *Plxdc2−/−* mice were challenged with 8% DSS (Fisher) in drinking water for 7 days in order to induce colitis. Mice were monitored daily through body weight change and assessing clinical signs of disease. Mice were euthanized at day 7 post-DSS challenge.

### Cell isolation and flow cytometry analysis

Excised colons and stomachs were opened and rinsed with 1 × PBS. Tissues were incubated 1 h at 37 °C stirring in RPMI media supplemented with Collagenase and DNAse. Samples were purified through a Percoll gradient. The interphase layer, corresponding to leukocytes, was collected and plated in 96-well plates for immunophenotype analysis. Single-cell suspensions from GLN were obtained as described above. Samples were then initially incubated with Fc block followed by a mixture of fluorochrome-conjugated antibodies against extracellular markers, including CD45, CD3, CD4, CD8, NK1.1, CD11b, CD64, F4/80, CD103, CD11c, MHC II, Ly6G, Ly6C. Samples were then incubated with Streptavidin-Texas Red for secondary staining. Samples were fixed and permeabilized (eBioscience) and incubated with the intracellular fluorochrome-conjugated antibody anti-TNFα. Data was acquired using a BD FACSCelesta flow cytometer instrument and analyzed using FACSDiva software (BD Bioscience).

### Histological analysis

Excised and cleaned colons and stomachs were fixed in 10% formalin. Colonic and gastric sections were paraffin embedded and Hematoxylin and eosin (H&E) stained. Slides were examined using and Olympus microscope. Images were obtained through Olympus CellSens Entry imaging software.

### Statistics

Data are expressed as mean values and standard error of the mean represented in error bars. To calculate significance of the RNAseq dataset, all genes with median expression level in all samples greater than 0 were included in a 3-way (genotype, treatment and time) ANOVA analysis. Normal quantile transformation (qqnorm from R^64^) was used to normalize the FPKM to fit the normality assumption of ANOVA (tested with Kolmogorov–Smirnov test). The 3-way ANOVA was carried in R^64^, FDR^65^ and Bonferroni were used to calculate the adjusted *P*-values. To determine significance of the standard data, Analysis of variance (ANOVA) was performed using the general linear model procedure in SAS (SAS Institute). Significance was considered at *P* ≤ 0.05 and significant differences were identified with an asterisk (genotype) or pound sign (infection or treatment).

## Supplementary information

Supplementary Information.

## Data Availability

The dataset generated during the current study is available in the NCBI Gene Expression Omnibus repository under the accession number GSE67270. Additional information is readily available from the corresponding author with reasonable request.
