# Supplementary material for: Identification of new regulatory genes through expression pattern analysis of a global RNA-seq dataset from a Helicobacter pylori co-culture system
Source: Sci Rep. 2020 Jul 13;10:11506. doi: 10.1038/s41598-020-68439-8 (PMC7359330; doi:10.1038/s41598-020-68439-8)
Supplement: Supplementary file 1 — Supplementary Information. [file 41598_2020_68439_MOESM1_ESM.pdf]

Identification of new regulatory genes through expression pattern analysis of a global RNA-seq dataset from a *Helicobacter pylori* co-culture system

Nuria Tubau-Juni[1,2], Josep Bassaganya-Riera[1,2], Andrew Leber[1,2], Victoria Zoccoli-Rodriguez[1,2], Barbara Kronsteiner[1], Monica Viladomiu[1], Vida Abedi[1], Casandra W. Philipson[1] and Raquel Hontecillas[1,2*]

[1] NIMML Institute (www.nimml.org), Blacksburg, VA, USA.

[2] BioTherapeutics, Inc, Blacksburg, VA, USA.

Correspondence: Dr. Raquel Hontecillas, NIMML Institute (www.nimml.org), Blacksburg, VA 24060, USA. E-mail: rmagarzo@nimml.org; phone: (540) 944-4197

Supplementary Information

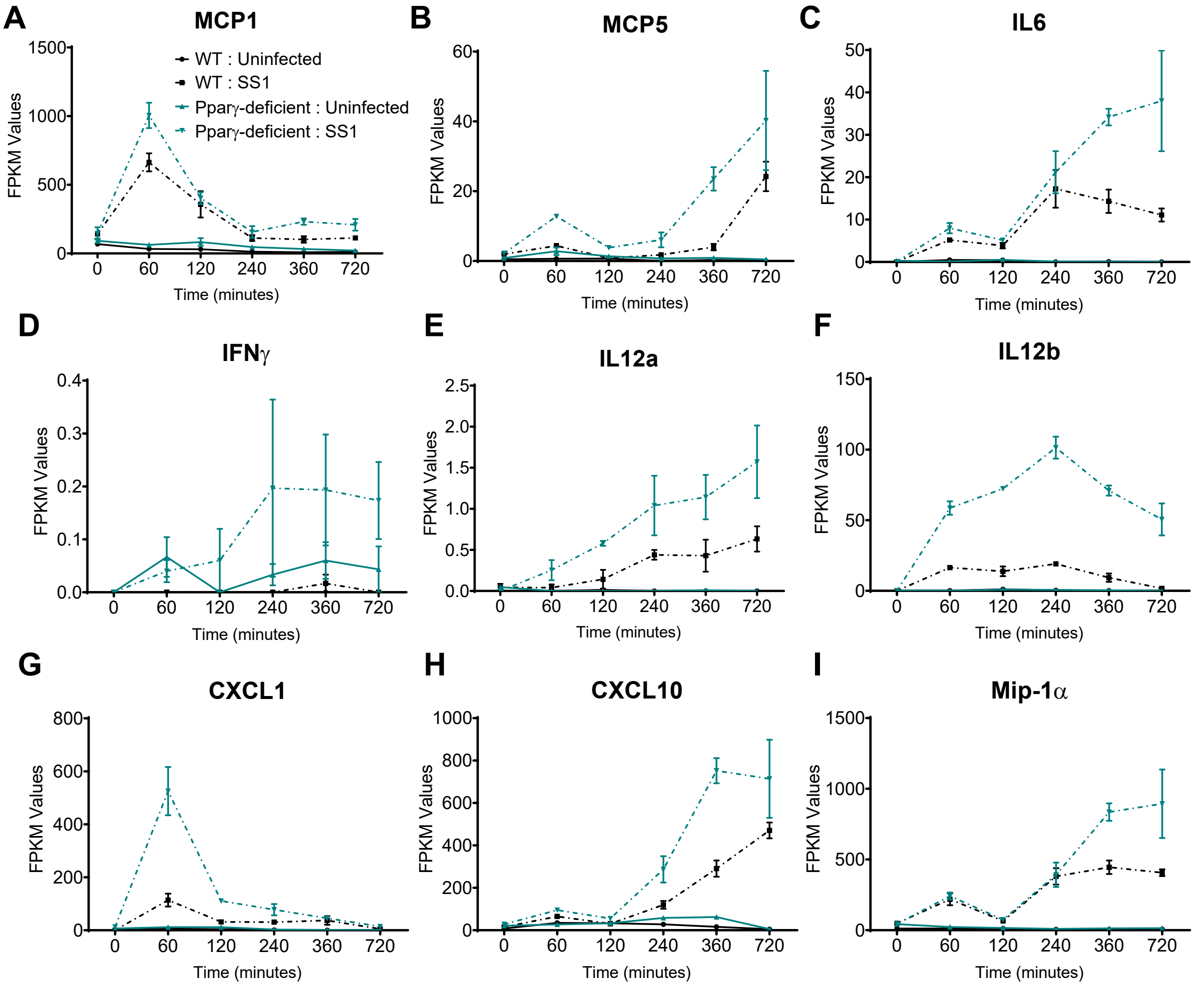

Supplementary Figure S1. Expression kinetics of several pro-inflammatory genes during *Helicobacter pylori* co-culture. Plots represent the RNAseq reads comparing WT and PPARγ-deficient BMDM at the indicated timepoints (min) of the gentamycin protection assay of *MCP1* (**A**), *MCP5* (**B**), *IL-6* (**C**), *IFNγ* (**D**), *IL12a* (**E**), *IL12b* (**F**), *CXCL1* (**G**), *CXCL10* (**H**), and *MIP-1α* (**I**).

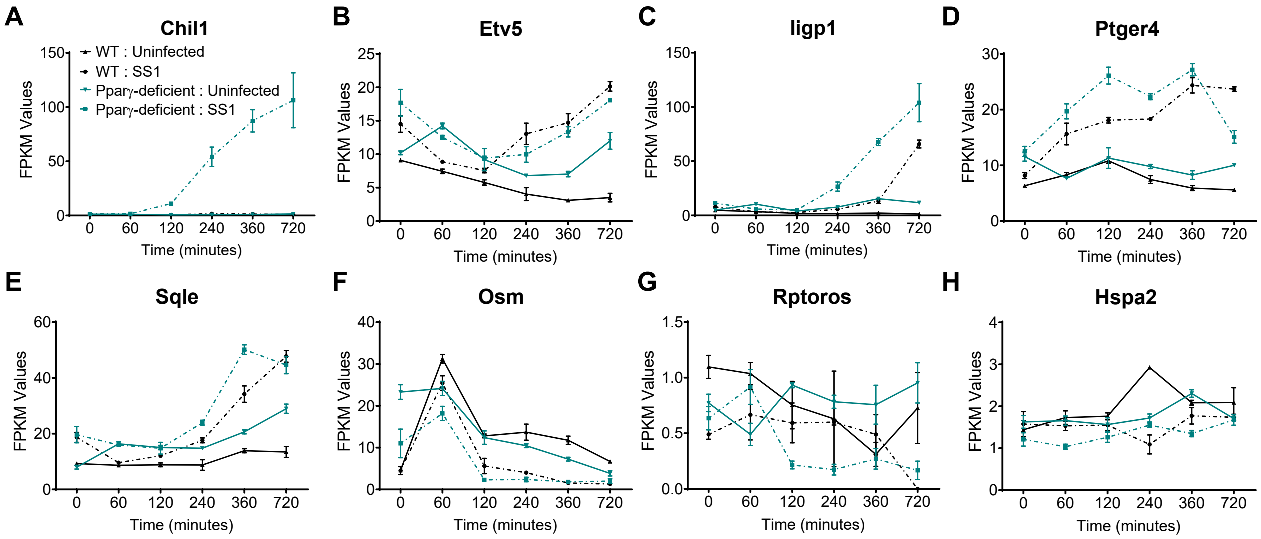

Supplementary Figure S2. 3-way ANOVA revealed 8 genes with *P < 0.05* at the interaction of treatment, genotype, and time. Plots represent the RNAseq reads of *Chil1* (**A**), *Etv5* (**B**), *Iigp1* (**C**), *Ptger4* (**D**), *Sqle* (**E**), *Osm* (**F**), *Rptoros* (**G**), and *Hspa2* (**H**) in WT and Pparγ-deficient BMDM during the entire time-course of the gentamycin protection assay.

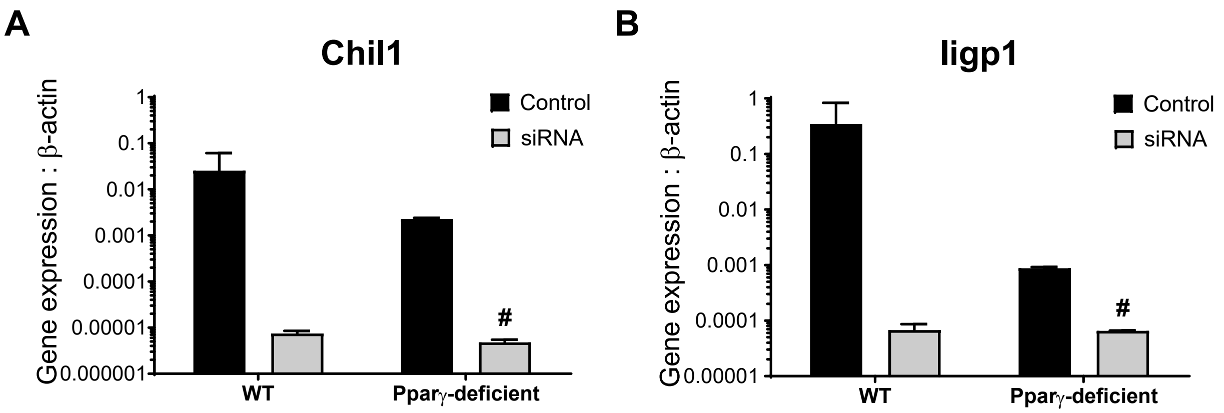

Supplementary Figure S3. Validation of *Chil1* and *Iigp1* gene silencing by qRT-PCR. WT and Pparγ-deficient macrophages were transfected with *Chil1*-targeted, *Iigp1*-targeted or negative scrambled siRNA prior to *H. pylori* challenge. Cells were harvested 120 min after *H. pylori* co-culture. *Chil1* (**A**) and *Iigp1* (**B**) gene expression was assessed. #*P*<0.05 within treatments.

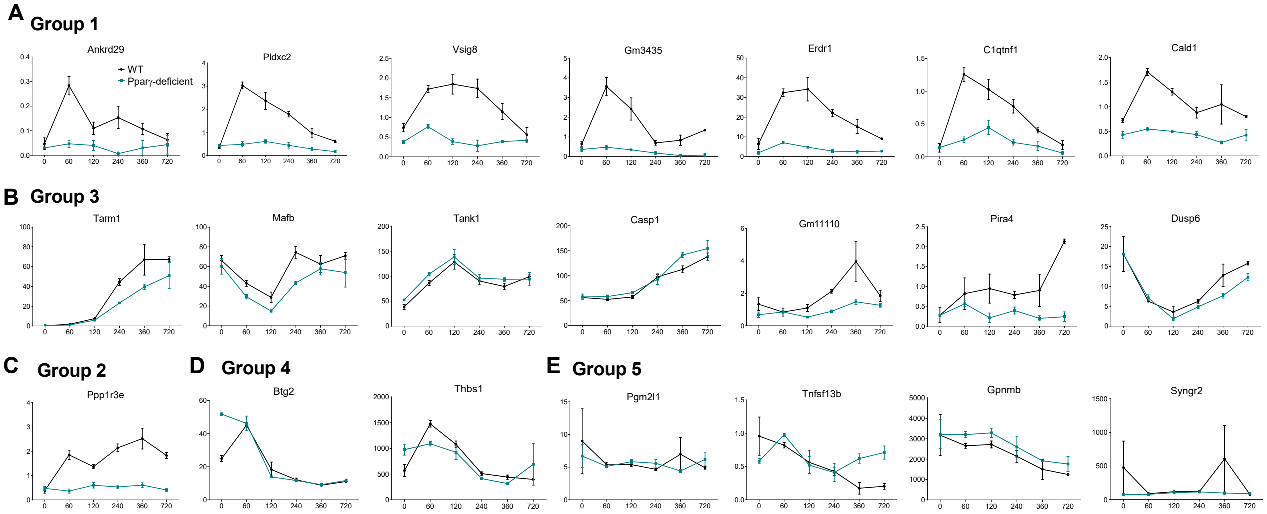

Supplementary Figure S4. **Final set of genes from the bioinformatics analysis with 21 candidates classified in five groups based on the expression kinetics.** Plots represent the RNAseq reads at each time point of the experiment comparing WT and PPARγ-deficient BMDM. Group 1 (**A**): *Ankrd29*, *Plxdc2*, *Vsig8*, *Gm3435*, *Erdr1*, *C1qtnf1*, and *Cald1*. Group 3 (**B**): *Term1*, *Mafb*, *Tank1*, *Casp1*, *Gm11110*, *Pira4*, and *Dusp6*. Group 2 (**C**): *Ppp1r3e*, whereas group 4 (**D**): *Btg2* and *Thbs1*. Group 5 (**E**): *Pgm2l1*, *Tnfsf13b*, *Gpnmb,* and *Syngr2*.

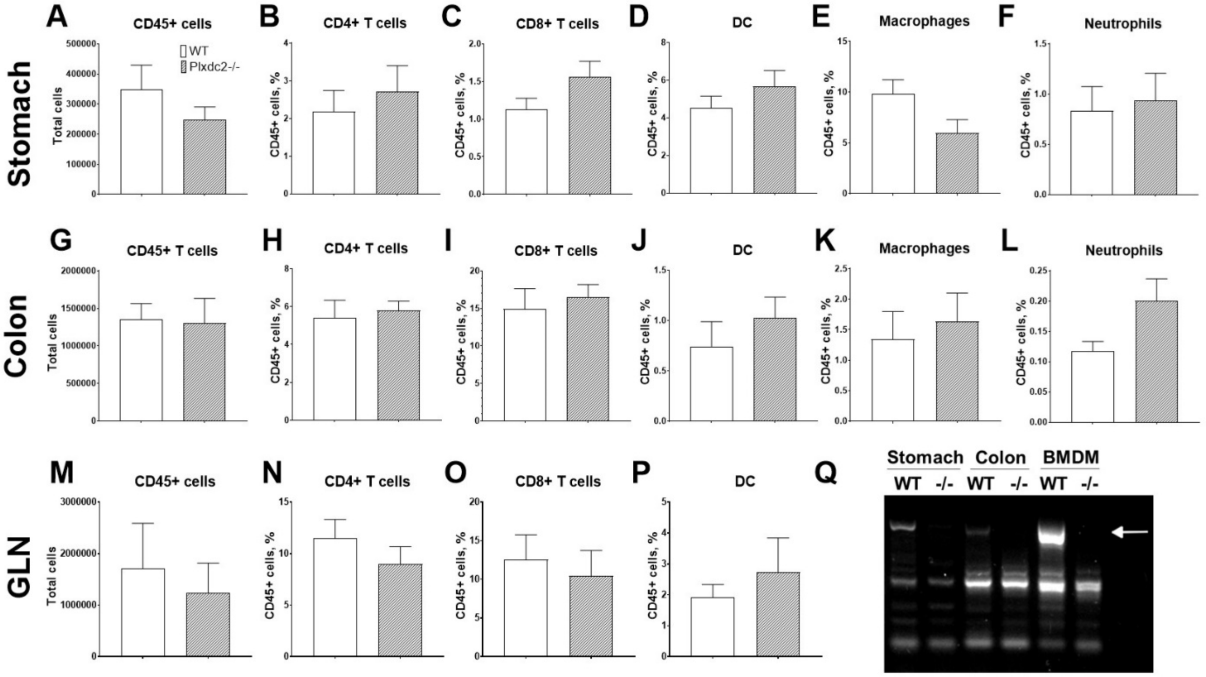

Supplementary Figure S5. **Phenotypic characterization of *Plxdc2*-deficient mice.** Single cell suspensions were obtained from stomach, colon and GLN for FACS analysis. Plots represent total number of CD45+ cells (**A, G, M**), and proportion of CD4+ T cells (**B, H, N**), CD8+ T cells (**C, I, O**), Dendritic cells (**D, J, P**), Macrophages (**E, K**), and Neutrophils (**F, L**). Detection of Plxdc2 gene expression in bulk mRNA isolated from stomach, colon and BMDM samples of WT and Plxdc2-/- mice (**Q**).

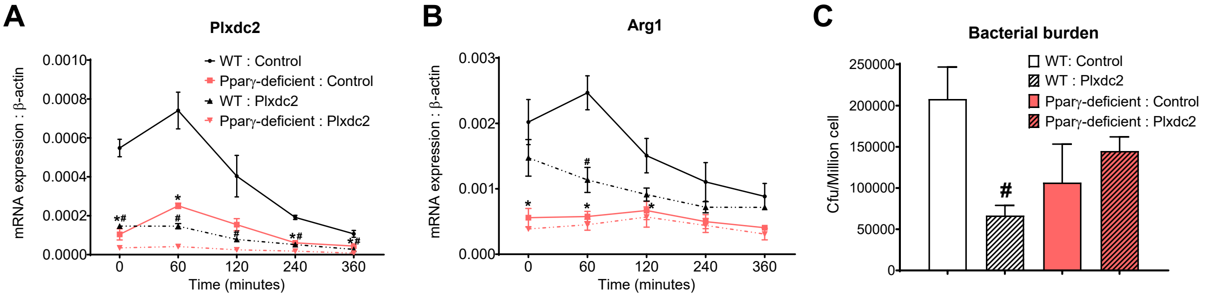

Supplementary Figure S6. Gene silencing studies to confirm the regulatory role of *Plxdc2.* WT and PPARγ-deficient BMDM were transfected with *Plxdc2* or scrambled siRNA as a negative control prior to *H. pylori* co-culture *in vitro*. Cells were harvested at 0, 60, 120, 240, and 360 min post *H. pylori* challenge and *Plxdc2* (**A**) *and Arg1* (**B**) gene expression were measured. Bacterial burden (**C**) was assessed 120 min post co-culture. **P*<0.05 between genotypes, #*P*-value<0.05 between treatment within each genotype.

Gene Symbol	Gene Full Name	Number of publications	Cellular Location	Function/Role	Ligands
Plxdc2	Plexin domain containing 2	6 (27)	Plasma membrane	PEDF receptor (IL-10 expression) [1]. Nervous system development [2,3]. Role in modulating coagulation [4].	PEDF
Ppp1r3e	Protein phosphatase 1, regulatory (inhibitor) subunit 3E	1	Cytoplasm	Glycogen metabolism (increases glycogen synthesis) [5].	None
Vsig8	V-set and immunoglobul in domain containing 8	2	Plasma membrane	Expressed in hair shaft, follicle, nail unit, and oral cavity [6]. VISTA receptor (potential target for cancer or infectious disease) [7].	VISTA (not commercialized)
Ankrd29	Ankyrin repeat domain 29	1	Nucleus	Niemann-Pick Type C diseases (lipid transport) [8].	None
C1qtnf1	C1q and tumor necrosis factor related protein 1	31 (36)	Cytoplasm, nucleus	Adipokyne related to glucose metabolism and diabetes [9,10]. Link to Pparγ (increased expression with rosiglitazone treatment) [11]. Role in cardiovascular diseases [12-14].	None

Supplementary Table 1. Gene information and properties of the 5 top selected candidates.

Gene	Forward Primer	Reverse Primer
β-Actin	CCGAGGCATTGCTGACAGG	TGGAAGGTCGACAGTGAGGC
Chil1	GCACCCACATCATCTACA	GACTCGTCATTCCACTCC
Iigp1	ACCTGCAAATTCTGTCTCA	TGTATGTCCATGTACCATATAAAC
Sqle	CGAAGTATACAGCCACATT	CTTCTTCATTCAGCCAACT
Plxdc2	TCTCCAGAGTCCAAAGGGTTCA	TTGTGATCCGTGTCCTCCTCTATC
Ppp1r3e	AAACAGCAGGTGTGCGTAAGT	AGGCTCCTCAGTCCATAAAGGT
Vsig8	TCTCCTACAAGTGGGCCAAGAT	GGTTGATGGTGCTGTGGAATGA
Ankrd29	GCAGTGCTCAGTGGGAATGTT	GGAGCTCATTGGCCTTGTTTCT
C1qtnf1	CACCATCCTGAAAGGCGAGAAAG	CCCTGGGACCTGTAGAACCTATTT
Il-10	GGGTTGCCAAGCCTTATCGGAAAT	TCTTCAGCTTCTCACCCAGGGAAT
Ifnγ	ATTAGCCAAGACTGTGAT	AGGTACAAGCTACAATCT
Arg1	GCCGATTCACCTGAGCTTTGAT	TCTGTAAGATAGGCCTCCCAGAAC
iNOS	GCTTTGTGCGAAGTGTCAGT	CTCCTTTGAGCCCTTTGTG
S100A8	GACATCAATAGTGACAAT	TATTCTGTAGACATATCCA
S100A9	CGACACCTTCCATCAATACTCT	TTGCCAACTGTGCTTCCA

Supplementary Table 2. qRT-PCR primers utilized in this study.

References

1 Cheng, G. *et al.* Identification of PLXDC1 and PLXDC2 as the transmembrane receptors for the multifunctional factor PEDF. *Elife* **3**, e05401, doi:10.7554/eLife.05401 (2014).

2 Miller, S. F. *et al.* Expression of Plxdc2/TEM7R in the developing nervous system of the mouse. *Gene Expr Patterns* **7**, 635-644, doi:10.1016/j.modgep.2006.12.002 (2007).

3 Miller-Delaney, S. F., Lieberam, I., Murphy, P. & Mitchell, K. J. Plxdc2 is a mitogen for neural progenitors. *PLoS One* **6**, e14565, doi:10.1371/journal.pone.0014565 (2011).

4 Thibord, F. *et al.* A Genome Wide Association Study on plasma FV levels identified PLXDC2 as a new modifier of the coagulation process. *J Thromb Haemost* **17**, 1808-1814, doi:10.1111/jth.14562 (2019).

5 Munro, S., Ceulemans, H., Bollen, M., Diplexcito, J. & Cohen, P. T. A novel glycogen-targeting subunit of protein phosphatase 1 that is regulated by insulin and shows differential tissue distribution in humans and rodents. *FEBS J* **272**, 1478-1489, doi:10.1111/j.1742-4658.2005.04585.x (2005).

6 Rice, R. H., Phillips, M. A. & Sundberg, J. P. Localization of hair shaft protein VSIG8 in the hair follicle, nail unit, and oral cavity. *J Invest Dermatol* **131**, 1936-1938, doi:10.1038/jid.2011.133 (2011).

7 Rosenzweig, M. M., Michael; Guo, Yalin; Rothstein, Jay. Identification of VSIG8 as the putative VISTA receptor and its use thereof to produce VISTA/VSIG8 modulators. US patent (2016).

8 Rodriguez-Pascau, L. *et al.* Characterisation of two deletions involving NPC1 and flanking genes in Niemann-Pick type C disease patients. *Mol Genet Metab* **107**, 716-720, doi:10.1016/j.ymgme.2012.10.004 (2012).

9 Xin, Y. *et al.* Elevated circulating levels of CTRP1, a novel adipokine, in diabetic patients. *Endocr J* **61**, 841-847, doi:10.1507/endocrj.ej14-0016 (2014).

10 Xin, Y. *et al.* C1qTNF-related protein 1 improve insulin resistance by reducing phosphorylation of serine 1101 in insulin receptor substrate 1. *Endocr J* **64**, 787-796, doi:10.1507/endocrj.EJ17-0128 (2017).

11 Wong, G. W. *et al.* Molecular, biochemical and functional characterizations of C1q/TNF family members: adipose-tissue-selective expression patterns, regulation by PPAR-gamma agonist, cysteine-mediated oligomerizations, combinatorial associations and metabolic functions. *Biochem J* **416**, 161-177, doi:10.1042/BJ20081240 (2008).

12 Chen, H. *et al.* C1qTNF-related protein 1 attenuates doxorubicin-induced cardiac injury via activation of AKT. *Life Sci* **207**, 492-498, doi:10.1016/j.lfs.2018.06.029 (2018).

13 Kim, D. & Park, S. Y. C1q and TNF related protein 1 regulates expression of inflammatory genes in vascular smooth muscle cells. *Genes Genomics* **41**, 397-406, doi:10.1007/s13258-018-0770-5 (2019).

14 Wang, H., Wang, R., Du, D., Li, F. & Li, Y. Serum levels of C1q/TNF-related protein-1 (CTRP-1) are closely associated with coronary artery disease. *BMC Cardiovasc Disord* **16**, 92, doi:10.1186/s12872-016-0266-7 (2016).
